# Multivariate connectivity deviations reveal task-evoked and clinical network reorganization

**DOI:** 10.1016/j.isci.2026.117959

**Published:** 2026-10-21

**Authors:** Jean-Philippe Kröll, Mohamed Abdelmotaleb, Harun Kocataş, Veronica Müller, Lya Paas, Marcus Meinzer, Agnes Flöel, Simon Eickhoff, Kaustubh R. Patil

**Affiliations:** 1 Institute of Neuroscience and Medicine, Brain & Behaviour (INM-7), Research Centre Julich, 52428 Julich, Germany; 2 Institute of Systems Neuroscience, Medical Faculty, Heinrich Heine University Dusseldorf, 40225 Dusseldorf, Germany; 3 Department of Neurology, University Medicine Greifswald, Greifswald, Germany; 4 German Centre for Neurodegenerative Diseases (DZNE) Standort Greifswald, Greifswald, Germany

**Keywords:** functional connectivity, normative modeling, one-class support vector machine, network neuroscience, multivariate analysis, task-induced connectivity reconfiguration, region-wise connectivity profiling, resting-state fMRI, early psychosis, perMANOVA

## Abstract

Understanding how cognitive demands and pathology reshape functional connectivity (FC) requires multivariate, region-specific methods. Here, we introduce one-class SVM-based connectome anomaly recognition (OSCAR), which detects condition-related deviations in regional connectivity profiles. OSCAR learns region-to-whole-brain connectivity patterns from a reference state using one-class support vector machines and quantifies target-condition deviations based on outlier proportions. We validated OSCAR across three diverse cognitive tasks, in which it identified regions within networks associated with conflict processing, object-location memory, and lexical learning. In a clinical cohort that included individuals with early psychosis, OSCAR identified relevant regions, including thalamic and basal ganglia areas. Compared with permutation-based multivariate analysis of variance, OSCAR detected additional established task- and disease-relevant parcels, while its task-related findings were at least as close, and often closer, to independent activation findings. OSCAR thus provides an interpretable, region-centered approach for mapping multivariate FC reconfigurations across experimental and clinical conditions.

## Highlights

• OSCAR is a new multivariate method for detecting regional connectivity deviations
• OSCAR identified relevant regions across three cognitive tasks and early psychosis
• OSCAR detected task- and disease-relevant parcels beyond those found by perMANOVA

## Graphical abstract

**Figure:**
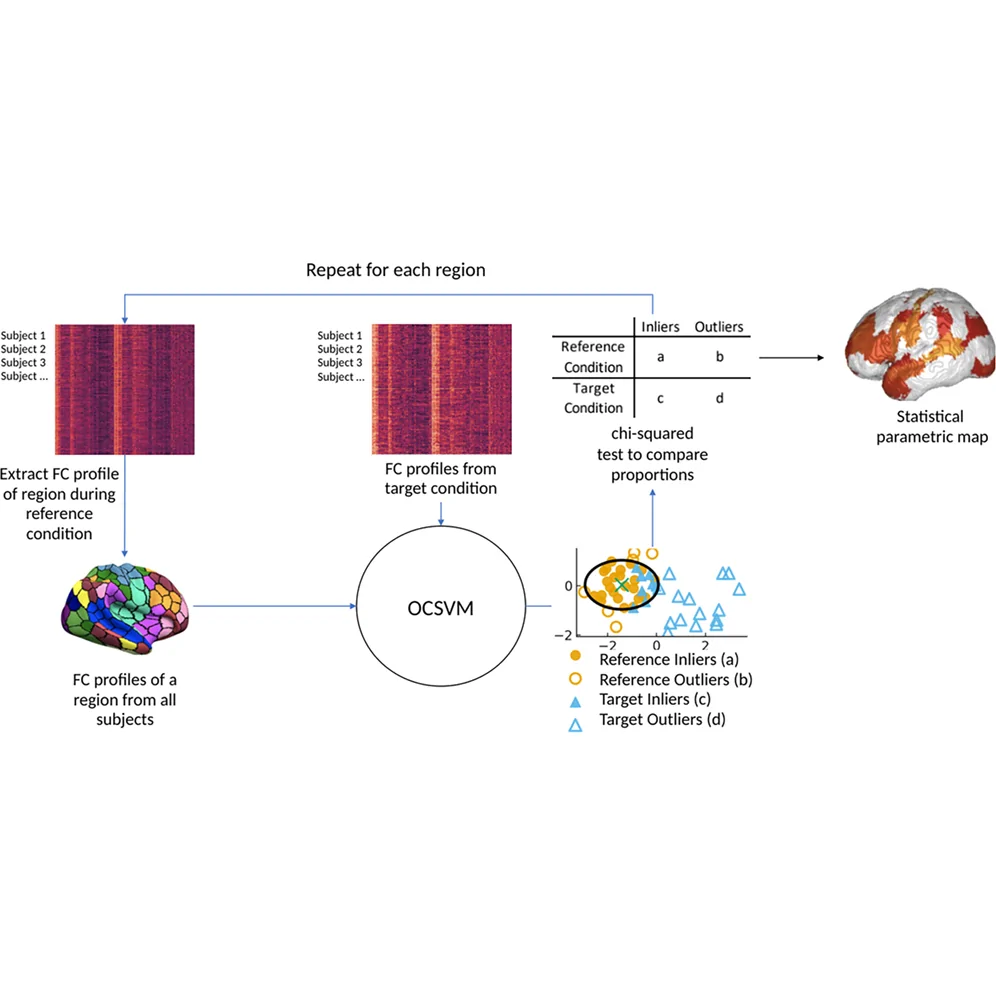

## Teaser

Applied computing in medical science; Applied sciences; Computer science; Health sciences; Internal medicine; Medical specialty; Medicine; Neurology

## Introduction

Understanding human brain function requires accounting for two fundamental organizational principles: regional specialization^1^ and functional integration.^2^ Regional specialization describes how distinct brain regions are tailored to support specific functions, such as sensory encoding,^3^ motor planning,^4^ memory formation,^5^ or abstract reasoning.^6^ Yet these localized functions alone cannot explain human cognition, and disease often manifested as coordination across many regions.^7^ Functional integration, therefore, refers to the context-dependent interactions between several brain regions. Contemporary neuroimaging work has shown that these principles are inherently intertwined: specialized regions contribute to cognition through their embeddedness within large-scale networks, while network-level dynamics depend on the differentiated capabilities of individual nodes.^8^

Crucially, this interplay is not static. The brain continually adapts its functional organization to meet the demands of unfolding cognitive processes, dynamically balancing between segregation and integration.^9^^,^^10^ These dynamics also imply that the functional organization can be (systematically) perturbed. Tasks or external inputs (such as naturalistic stimuli) place distinct demands on regional processing, and any local change is expected to drive corresponding changes in large-scale connectivity. Conversely, altered connectivity necessarily modifies the functional role of individual regions. Understanding how brain organization is modulated by a given context is critical for elucidating the neural basis of behavior as well as clinical conditions. The present work proposes a new method to detect changes in brain functional organization based on how each region is typically embedded within whole brain networks.

While both activation-based and conventional univariate functional connectivity (FC) analyses can reveal condition-specific effects, each captures only part of the underlying reconfiguration.^11^^,^^12^ Activation studies focus on local signal changes but do not describe how these local effects alter a region’s embedding within large-scale networks. Univariate FC analyses, in turn, capture changes in individual connections but do not consider multivariate differences. Methods to identify multivariate FC differences have been proposed.^13^^,^^14^^,^^15^ However, these methods are designed to compare conditions directly, rather than to characterize alterations relative to a learned reference distribution. On the other hand, data-driven approaches such as independent component analysis (ICA) provide information about large-scale functional networks, but they do not directly test whether specific regions alter their connectivity patterns in a condition-dependent manner.^16^^,^^17^ Emerging approaches complement these methods by modeling a region’s connectivity patterns from a reference state and then quantify deviations in a target state yielding spatially specific and context-sensitive reorganization. These frameworks thus have the potential to uncover reorganizations that may be subtle, distributed, or not well captured by other methods. Thereby, these approaches can help characterize how tasks reorganize network structure and how external perturbations, such as task involvement and disease status, modify these networks, even under heterogeneous or less-controlled conditions. A recent review by Toloknieiev et al.^18^ identified five publications proposing such metrics of abnormal connectivity quantification. However, these methods either operate at a voxel level^19^^,^^20^ or have only been applied to diseases, limiting their generalizability.^21^^,^^22^^,^^23^

We here propose a new method, one-class SVM-based connectome anomaly recognition (OSCAR), that uses a sophisticated multivariate density estimation method (OCSVM), and considers region-to-whole brain patterns of connectivity. It provides a *p* value for each region that can be used to identify regions whose multivariate connectivity pattern differs in the target condition compared with the reference. We here consider the identification of regions affected by a given target condition an outlier detection problem, treating healthy resting state (RS) as the reference state and aiming to detect outliers within several tasks and patient data. To contextualize OSCAR findings on the different tasks, we compared them with activation-based analyses, which remain the standard approach for identifying task-relevant brain regions. However, because local activation and FC capture related but distinct aspects of brain organization,^12^^,^^24^ we did not expect OSCAR findings to fully overlap with activation-based results. Instead, we expected OSCAR to partly confirm known task-related regions while also revealing additional connectivity-based network reconfigurations. We applied OSCAR to the data from the Amsterdam Open MRI Collection (AOMIC, RS, and Gender-Stroop task)^25^ and two task datasets from the *Modulation of brain networks for learning and memory by transcranial electrical brain stimulation: A systematic, lifespan approach* (MeMoSLAP: https://www.memoslap.de/de/forschung/). Here, we expected OSCAR to identify task-relevant regions, based on the assumption that regions engaged by a given task should exhibit changes in their connectivity profiles. Further, we tested OSCAR on data from patients with early psychosis (EP) from the Human Connectome Project for Early Psychosis (HCP-EP).^26^ Based on previous findings that psychosis is accompanied by a disruption in RS FC (rsFC),^27^^,^^28^ we anticipated that OSCAR would detect psychosis-specific FC abnormalities that diverge from the FC patterns observed in healthy individuals. We compared our results to a standard multivariate analysis of variance based on permutation testing (perMANOVA).^29^

## Results

To provide anatomical interpretations of Schaefer (and Tian) parcels, we mapped each parcel to the Harvard-Oxford (HO) cortical and subcortical atlases^30^ by computing voxelwise spatial overlap in MNI space. For each parcel, the HO region with the highest overlap fraction was assigned as its anatomical match. In the following, we report Schaefer/Tian parcels as well as the corresponding HO label.

### RS vs. GStroop task

For the GStroop task, out of 232 regions, OSCAR identified 110 regions, while perMANOVA found 56 regions to be significantly different (Figure 1). OSCAR and perMANOVA shared 32 regions (Figure 1A). The right-hemisphere precuneus (Schaefer: RH_ContC_pCun_2, HO: Precuneous Cortex), the right anterior globus pallidus (Tian: aGP-rhm, HO: Right Pallidum), and part of the left-hemisphere somatomotor A network (Schaefer: LH_SomMotA_3, HO: Postcentral Gyrus) were the three most significantly different regions found by OSCAR (Table S1). The perMANOVA identified a parcel of the right-hemisphere dorsal attention network’s frontal eye fields and two parcels in the ventral prefrontal cortex of the left hemisphere, belonging to the default network (Table S2). Spatial correspondence between the findings of both methods was on average low, with nearest-neighbor distances ranging from 0 to 50 mm (mean = 13.5 mm) (Figure 2B). For the negative GStroop contrast (Figure 2C), the spatial correspondence between regions identified by the two methods and the nearest activation peak did not differ significantly. For this contrast, both OSCAR and perMANOVA showed five regions (of 56 total, 8.9%) located more than 14 mm away from the nearest activation peak. On the other hand, for the positive GStroop contrast (Figure 2D), OSCAR-identified regions were significantly closer to the respective activation peaks, indicating a stronger spatial correspondence relative to perMANOVA (Mann-Whitney U test, *p* = 0.00349). OSCAR showed fewer regions located more than 14 mm away from the nearest activation peak (2/56 regions; 3.6%) compared with perMANOVA (11/56 regions; 19.6%).

**Figure 1:**
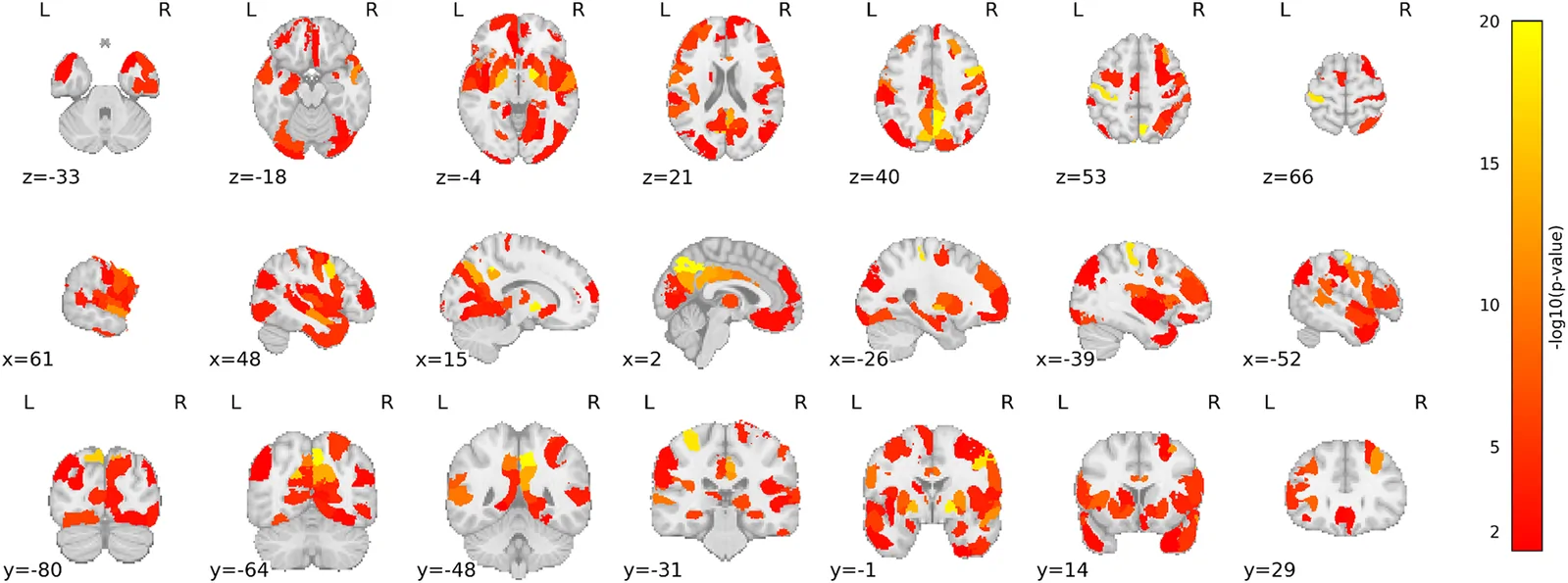
Brain regions with significantly different connectivity patterns between RS and the GStroop task, as identified by OSCAR L, left; R, right; *x*, sagittal; *y*, coronal; *z*, axial.

**Figure 2:**
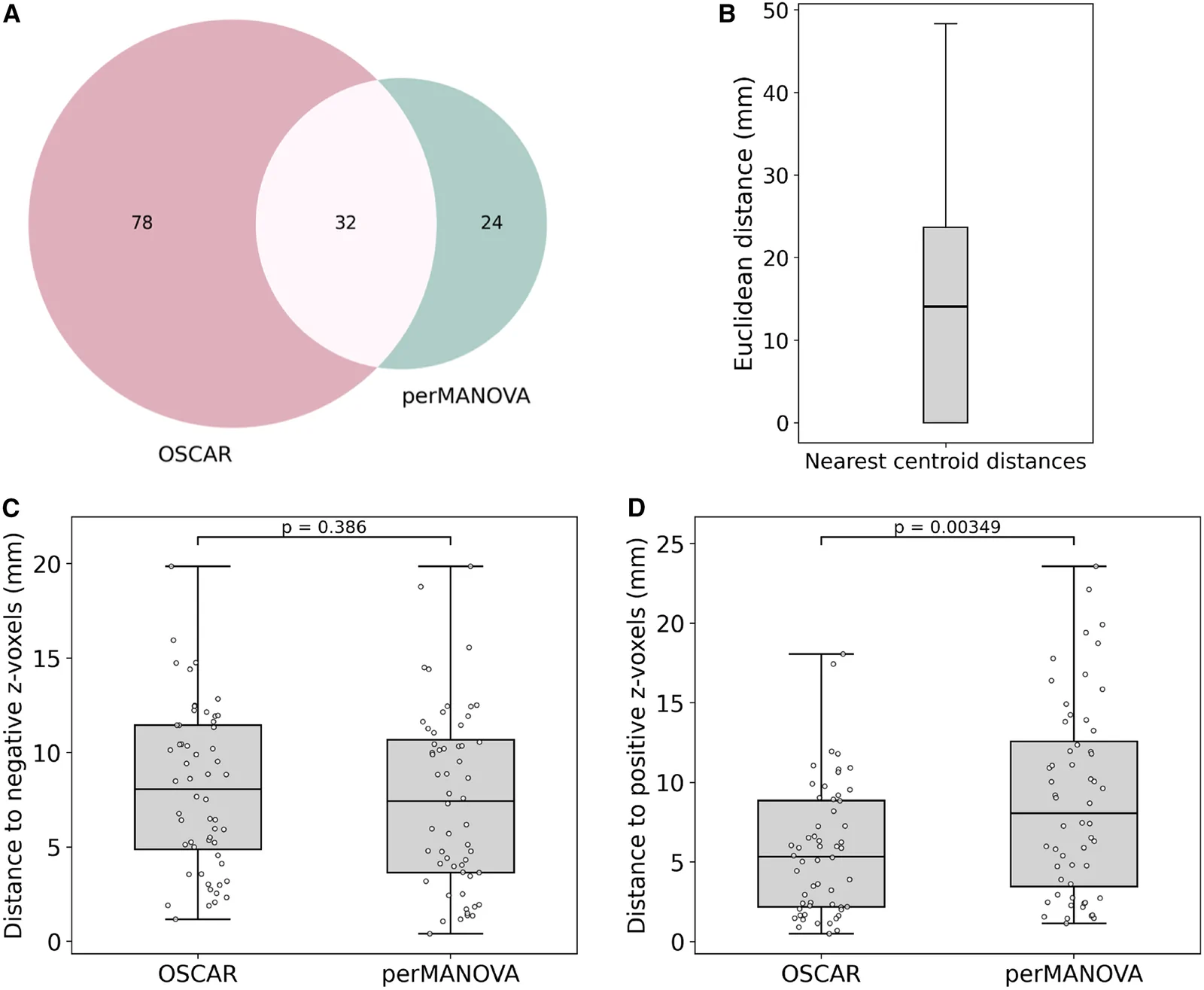
Comparison between OSCAR and perMANOVA results for the GStroop task (A) Overlap between significant regions identified by OSCAR and perMANOVA. The left circle (pink) represents the 78 regions detected only by OSCAR, the right circle (green) represents the 24 regions only identified by perMANOVA, and the intersection shows the 32 regions jointly identified by both methods. (B) Spatial correspondence between the two methods. The plot displays the minimum Euclidean distance from each region identified by one method to the nearest region identified by the other. (C) Euclidean distance from each region’s centroid to the nearest activation peak in the negative GStroop contrast (correct > incorrect), with shorter distances indicating greater spatial correspondence. (D) Euclidean distance from each region’s centroid to the nearest activation peak in the positive GStroop contrast (incorrect > correct), with shorter distances indicating greater spatial correspondence. Boxplots show the median (center line), interquartile range (box), and values within 1.5 × the interquartile range (whiskers).

### RS vs. OLM task

OSCAR identified 48 regions with deviating connectivity in the object-location memory task (OLM) task compared to RS (Figure 3). The perMANOVA found 37 significantly differing regions. OSCAR and perMANOVA shared 18 regions (Figure 4A). The right-hemisphere visual peripheral network’s extrastriate inferior (Schaefer: RH_VisPeri_ExStrInf_1, HO: Lingual Gyrus), striate calcarine (Schaefer: RH_VisPeri_StriCal_1, HO: Intracalcarine Cortex), and occipital pole (Schaefer: RH_VisCent_ExStr_5, HO: Occipital Pole) regions showed the most pronounced state-dependent changes according to OSCAR (Table S3).

**Figure 3:**
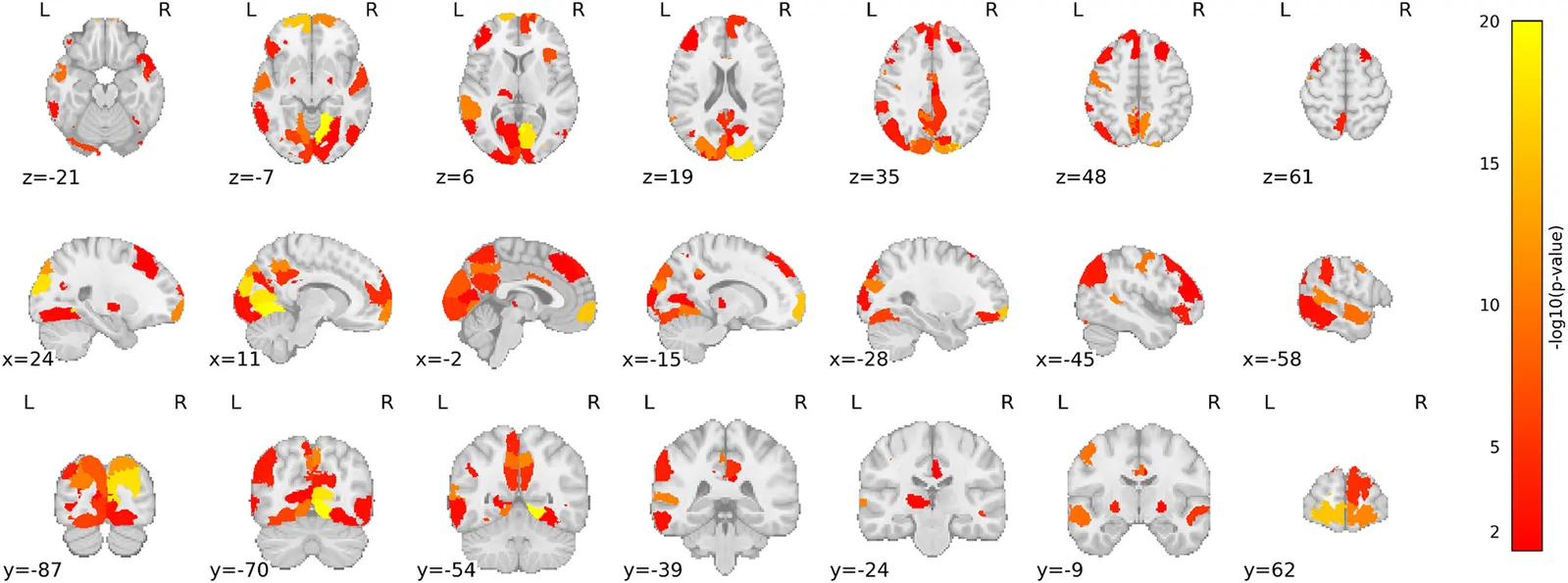
Brain regions with significantly different connectivity patterns between RS and the OLM-task, as identified by OSCAR L, left; R, right; *x*, sagittal; *y*, coronal; *z*, axial.

**Figure 4:**
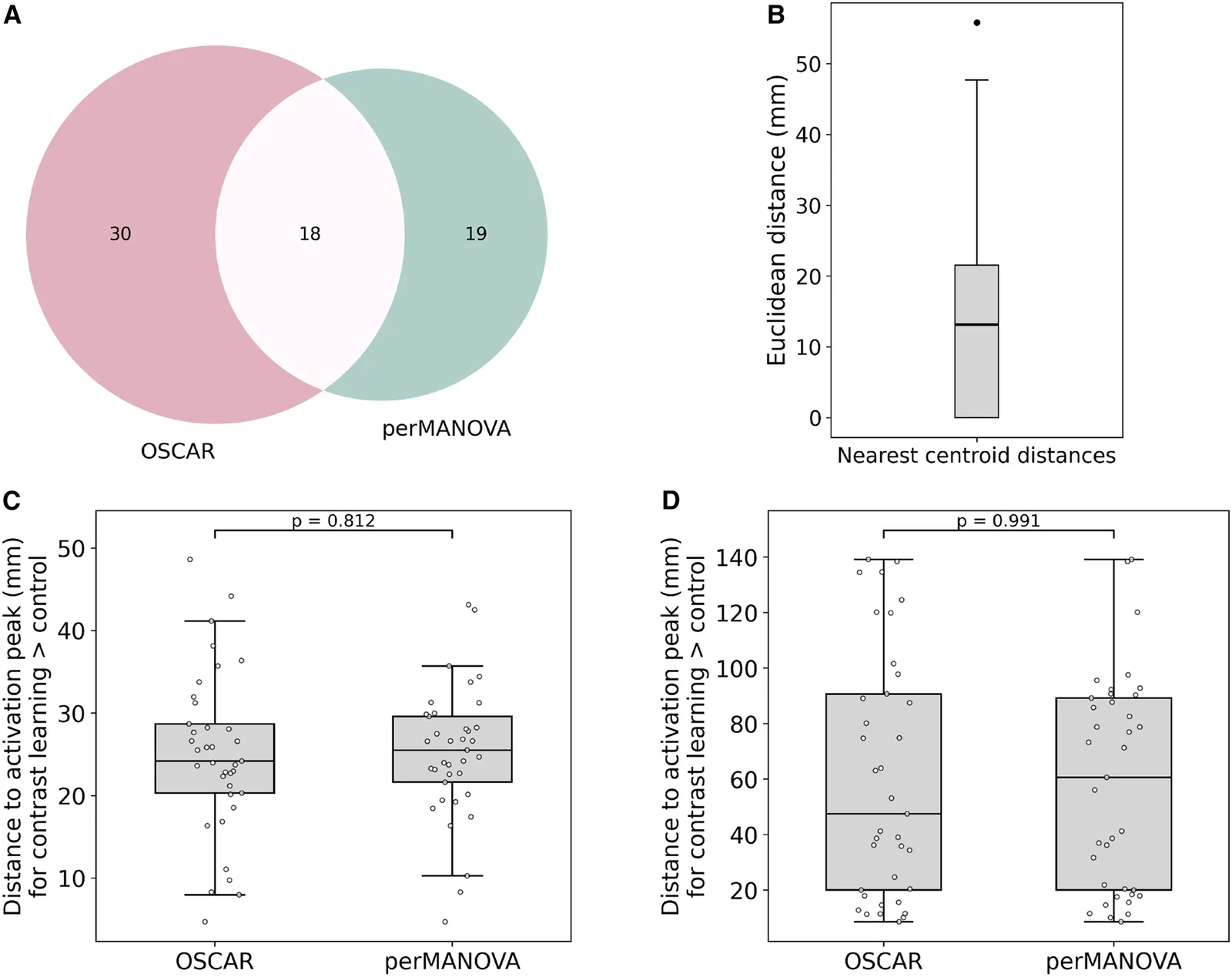
Comparison between OSCAR and perMANOVA results for the OLM task (A) Overlap between significant regions identified by OSCAR and perMANOVA. The left circle (pink) represents the 30 regions detected only by OSCAR, the right circle (green) represents the 19 regions only identified by perMANOVA, and the intersection shows the 18 regions jointly identified by both methods. (B) Spatial correspondence between the two methods. The plot displays the minimum Euclidean distance from each region identified by one method to the nearest region identified by the other. (C) Euclidean distance from each region’s centroid to the nearest activation peak in the OLM task for the contrast learning > control, with shorter distances indicating greater spatial correspondence. (D) Euclidean distance from each region’s centroid to the nearest activation peak in the OLM task for the contrast control > learning, with shorter distances indicating greater spatial correspondence. Note that the control > learning contrast only includes five regions. Boxplots show the median (center line), interquartile range (box), and values within 1.5 × the interquartile range (whiskers).

The perMANOVA identified the left planum porale (Schaefer: LH_SomMotB_Aud_1, HO: Planum Porale), the cingulate gyrus (Schaefer: RH_SalVentAttnA_ParMed_1, HO: Cingulate Gyrus, posterior division), and the central opercular cortex (Schaefer: LH_SomMotB_S2_2, HO: Central Opercular Cortex) as the three most significant regions (Table S4). Spatial correspondence between the findings of both methods was moderate, with nearest-neighbor distances ranging from 0 to >50 mm (mean = 13.9 mm) (Figure 4B). For both contrasts (learning > control and control > learning), OSCAR-identified regions were on average closer to the respective activation peaks, although not reaching significance (Figures 4C and 5D). For the learning > control contrast, the number of regions located more than 14 mm away from the nearest activation peak were 32/37 (86.5%) for OSCAR and 34/37 (91.89%) for perMANOVA. For the control > learning contrast, the number of regions located more than 14 mm away from the nearest activation peak were 31/37 (83.8%) for OSCAR and 33/37 (89.19%) for perMANOVA.

**Figure 5:**
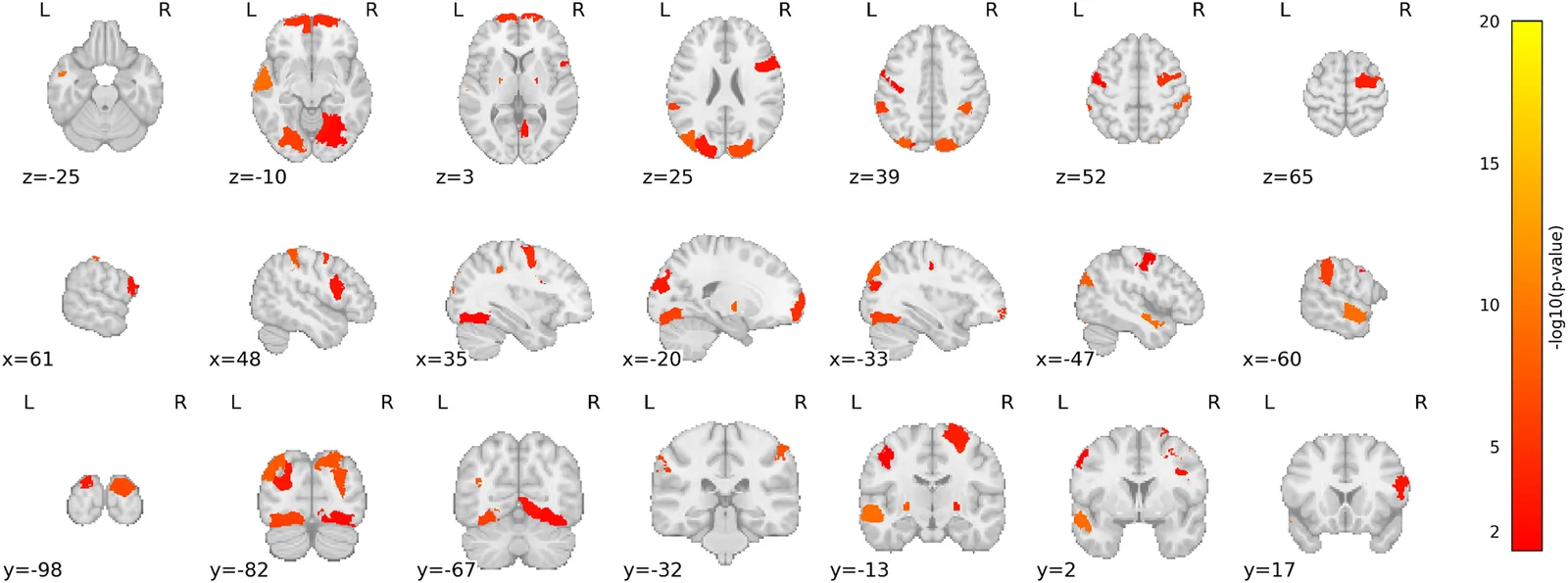
Brain regions with significantly different connectivity patterns between RS and the NWL task, as identified by OSCAR L, left; R, right; *x*, sagittal; *y*, coronal; *z*, axial.

### RS vs. NWL task

For the novel-word-learning (NWL) task, OSCAR identified 18 regions with deviating connectivity; perMANOVA found 17 significantly different regions (Figure 5). OSCAR and perMANOVA shared three regions (Figure 6). OSCAR showed highest significance in the left-hemisphere superior temporal gyrus (Schaefer: LH_DefaultB_Temp_3, HO: superior temporal gyrus, anterior division), the left globus pallidum (Schaefer: pGP-lh, HO: left pallidum), and the lateral occipital cortex (Schaefer: LH_DefaultC_IPL_1, HO: lateral occipital cortex, superior division, (Table S5). The perMANOVA identified the superior temporal gyrus (Schaefer: LH_DefaultB_Temp_3, HO: superior temporal gyrus, anterior division), the precuneus cortex (Schaefer: RH_DorsAttnB_PostC_3, HO: Precuneous Cortex), and lateral occipital cortex (Schaefer: RH_VisPeri_ExStrSup_3, HO: lateral occipital cortex, superior division) as the most significant regions (Table S6). Spatial correspondence between the findings of both methods was low, with nearest-neighbor distances ranging from 0 to >50 mm (mean = 22.8 mm) (Figure 6B). Both methods exhibited similarly low proximity to the activation peaks of the meta-analysis (Figure 6C) as almost all identified regions by both methods were more than 14 mm away from the nearest activation peak.

**Figure 6:**
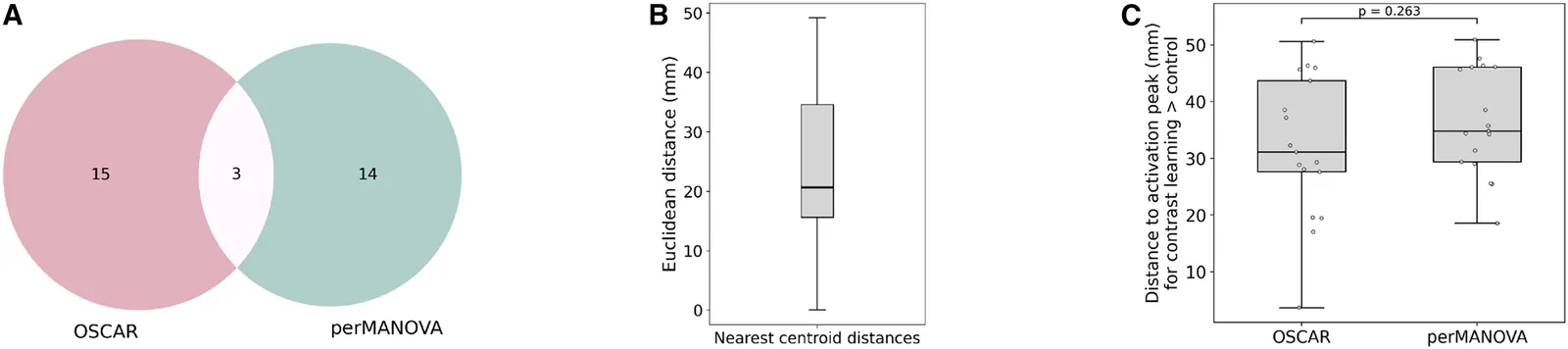
Comparison between OSCAR and perMANOVA results for the NWL task (A) Overlap between significant regions identified by OSCAR and perMANOVA. The left circle (pink) represents the 15 regions detected only by OSCAR, the right circle (green) represents the 14 regions only identified by perMANOVA, and the intersection shows the 3 regions jointly identified by both methods. (B) Spatial correspondence between the two methods. The plot displays the minimum Euclidean distance from each region identified by one method to the nearest region identified by the other. (C) Euclidean distance from each region’s centroid to the nearest peak coordinates from the meta-analysis on lexical learning, with shorter distances indicating greater spatial correspondence. Boxplots show the median (center line), interquartile range (box), and values within 1.5 × the interquartile range (whiskers).

### RS of healthy controls vs. RS of EP patients

On data from patients with EP, OSCAR identified 10 regions with deviating RS connectivity compared to RS from healthy controls (Figure 7). The perMANOVA found 3 significantly different regions, all of which were also identified by the OSCAR. OSCAR showed highest significance in the right-hemisphere ventral anterior thalamus (Tian: THA-VA-rh, HO: Right Thalamus), the left-hemisphere dorsal anterior thalamus (Tian: THA-DA-lh, HO: Left Thalamus), and the right posterior globus pallidus (Tian: pGP-rh, HO: Right Pallidum) (Table S7). The three regions identified by the perMANOVA were the left-hemisphere dorsal anterior thalamus, the right-hemisphere dorsal anterior thalamus, and the left-hemisphere ventral anterior thalamus (Table S8).

**Figure 7:**
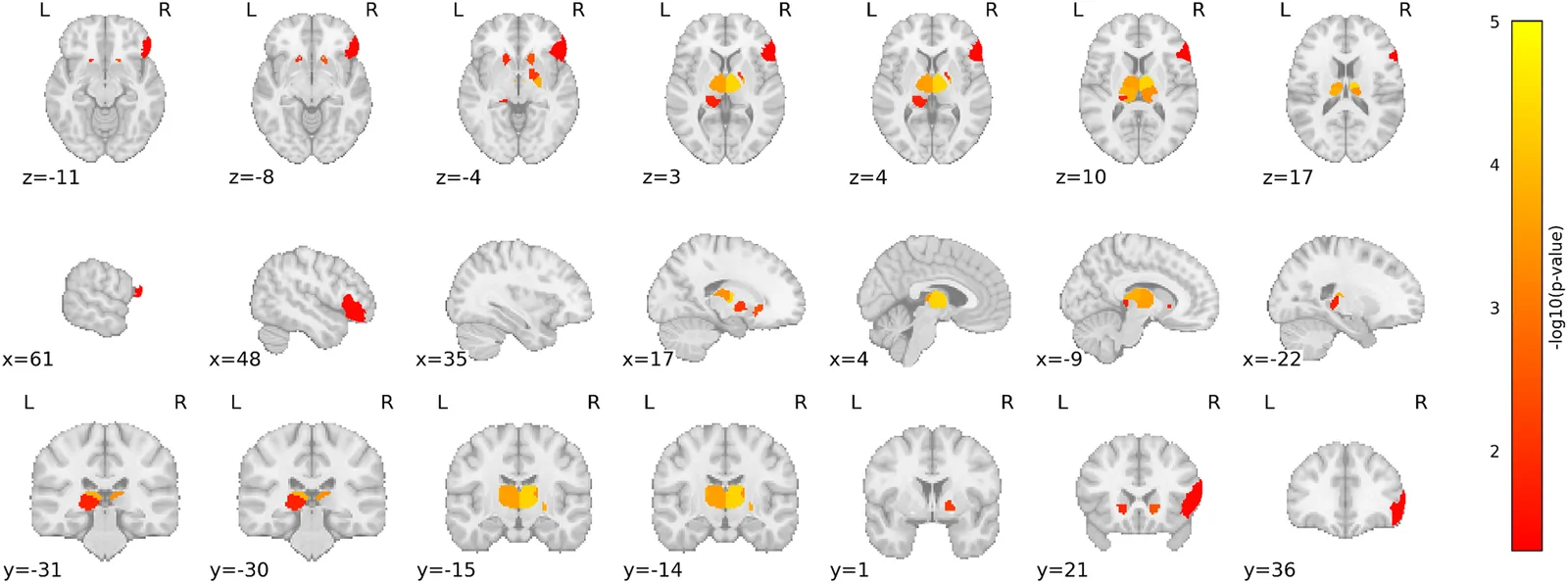
Brain regions with significantly different connectivity patterns between RS from healthy controls and RS from patients with early psychosis, as identified by OSCAR L, left; R, right; *x*, sagittal; *y*, coronal; *z*, axial.

## Discussion

We here proposed and validated a new method termed OSCAR to identify multivariate group differences in FC patterns of brain regions. Permutation-based MANOVA was employed as the baseline method for comparison.

First, OSCAR and perMANOVA were applied to FC data from three different tasks across three healthy cohorts: gender stroop, OLM, and NWL. In all three tasks, both methods detected shared and unique regions whose whole-brain connectivity significantly differed between RS and task state. The unique regions identified by OSCAR often included regions previously established as task relevant in the literature, which perMANOVA failed to identify. For instance, in GStroop task (e.g., insula and anterior cingulate cortex), for OLM (e.g., bilateral Pallidum and left Thalamus), and for NWL (e.g., medial prefrontal cortex and bilateral fusiform gyrus), several task-related regions were identified only by OSCAR.

Applied to the GStroop task, both methods identified several regions that are known to be involved in the processing of the (Gender-) Stroop task such as the precuneus,^31^^,^^32^ the posterior cingulate gyrus,^31^^,^^32^ the precentral gyrus,^33^ and the lateral prefrontal cortex.^34^ In addition, OSCAR detected connectivity differences in 78 regions that were not detected by perMANOVA (Figure 1). Notably, only OSCAR identified the insula and the anterior cingulate cortex, both involved in conflict monitoring during the Stroop task.^35^^,^^36^^,^^37^ OSCAR also detected several other regions known to be involved in the Stroop task such as the caudate,^38^ parts of the thalamus,^39^ and the inferior frontal gyrus.^36^

Applied to data from the OLM task, both methods identified connectivity differences in several regions that are a part of the ventral visual stream that is necessary for object identification^40^^,^^41^ and play a prominent role during the OLM task. Further, OSCAR and perMANOVA identified the precuneus, the retrospenial cortex, the middle frontal gyrus, the insula cortex, and the precentral gyrus all of which are known to be related to visual spatial learning.^42^^,^^43^^,^^44^ In addition, both methods identified the lateral occipital complex, which is tied to object recognition, object form perception, and object naming.^43^^,^^45^ Only OSCAR identified connectivity changes in further regions that have been indicated to be involved in OLM tasks by Gillis et al. such as the bilateral Pallidum, and the left thalamus.^43^ In addition, OSCAR identified the frontal orbital cortex, which might reflect feedback-based learning and decision-making,^46^ and parts of the left hemisphere inferior and middle temporo-occipital cortices, which are involved in object processing^41^^,^^43^ (Figure 3). Notably, the regions identified by OSCAR largely overlap with a general linear model analysis from a recent publication on the same data by Abdelmotaleb et al.^47^ Compared with the significant clusters of task activity found by Abdelmotaleb et al., OSCAR-identified regions were closer than perMANOVA on average, although not statistically significantly (Figures 4C and 4D).

For the NWL task, both methods detected the left superior temporal gyrus, which is a core region for phonological processing, speech perception, and lexical access.^48^^,^^49^ In addition, both methods identified changes in the extra-striate superior, which might reflect the visual component of the task stimulus. OSCAR additionally identified connectivity changes in the medial prefrontal cortex, which supports the integration of new information into existing schemas, as required when linking pseudowords to familiar objects in the NWL task^50^^,^^51^^,^^52^ and bilateral fusiform gyrus, which has been demonstrated to be involved in lexical learning.^53^ Further, OSCAR detected changes in the globus pallidus, which is tied to language functions,^54^ and the orbito-frontal cortex, which showed a significant main effect of learning stage during the NWL task in Sliwinska et al.^55^ (Figure 5). Notably, OSCAR did not detect the inferior frontal gyrus (IFG), left supplementary area, or insula, all of which have reliably shown strong effects in previous activation studies.^53^^,^^55^^,^^56^ To further investigate this discrepancy, we conducted an additional parcel-wise GLM analysis on the same dataset (Table S10). This analysis revealed significant task-evoked activity in 20 regions, among them the left IFG, left supplementary motor area, and insula. Possibly, the task-evoked activity in these regions does not translate into widespread connectivity reconfiguration. Instead, changes may be confined to a few specific connections and thus remain undetected when focusing on differences in region-to-whole-brain connectivity profiles as is done by OSCAR. Further, conventional task-based fMRI analyses model the time-locked structure of stimulus presentation using event-related or block-design regressors, enabling detection of transient, spatially localized responses. Such effects may be present only briefly and, when connectivity is aggregated across time, fail to produce sustained or distributed changes in region-to-whole-brain connectivity detectable by OSCAR.

Additionally, in a disease-related cohort, OSCAR likewise identified connectivity differences in disease-related regions that are known to be affected in EP not identified by a perMANOVA. Both methods identified three thalamic regions, the dorsal anterior subregion on both hemispheres and the ventral anterior subregion on the left hemisphere. OSCAR also identified differences in additional parts of the thalamus, the nucleus accumbens, the globus pallidus, as well as the ventromedial prefrontal cortex, all of which are known to be affected in psychosis^57^^,^^58^^,^^59^^,^^60^ (Figure 7). These results align with the previous literature as the thalamus has long been associated with psychosis and is believed to play a key role in its pathophysiology, affecting sensory, cognitive, and sleep processes.^61^^,^^62^ Taken together, those results show the utility of OSCAR in reliably identifying multivariate changes in FC patterns.

We also calculated the Euclidean distance between the centroids of the identified regions with differing FC and the peak coordinates from the corresponding task-activation analyses. Although condition-dependent changes in FC and task-evoked activity reflect distinct aspects of brain function, assessing their spatial correspondence provides a useful benchmark for evaluating the proposed method. Specifically, spatial proximity between task-activation peaks and regions exhibiting connectivity changes may indicate a convergence of network reconfiguration and regional task engagement. For the GStroop and OWL tasks, both OSCAR and perMANOVA identified regions located within 10 mm of task-activation peaks. However, across all three tasks—particularly the NWL task—several identified regions were situated substantially farther from the activation peaks. This supports the premise that connectivity reconfiguration and task-evoked activity are only partially overlapping phenomena. Overall, regions identified by OSCAR were generally closer to activation peaks than an equivalent number of regions identified by perMANOVA, though this difference was statistically significant only for the positive GStroop contrast (incorrect > correct; Figure 2D). While spatial proximity is only an indirect measure of correspondence, these findings indicate that a subset of regions exhibiting condition-dependent connectivity changes spatially aligns with those strongly engaged during task execution.

Taken together, we demonstrated here that OSCAR can identify regions where FC profile deviates due to the task engagement or a disease state. Importantly, OSCAR extended the findings of a standard perMANOVA by localizing effects in additional condition-related regions, suggesting that OSCAR is more sensitive to the multivariate effects induced by different conditions. OSCAR captures the multivariate connectivity pattern of a given region during the reference state and then identifies deviations due to a condition. In contrast, perMANOVA tests whether the mean connectivity pattern (the “centroid”) between the two groups differs spatially.^29^ Thus perMANOVA is sensitive to the dispersion of points, but it cannot detect deviations in the distribution of the data.^29^ Therefore, OSCAR might be more sensitive to subtle differences in FC patterns induced by a task or disease. Beyond its empirical performance, OSCAR also provides a conceptual contribution to the analysis of condition-related FC. Traditional task-based activation analyses identify localized changes in BOLD amplitude and thus reveal where neural responses increase or decrease during a task.^63^ However, activation maps do not capture whether the pattern of connectivity between a region and the rest of the brain changes.^64^ OSCAR quantifies how a region’s multivariate connectivity profile deviates across states, allowing researchers to detect condition-relevant connectivity reconfigurations, which might not be accompanied by local activation. Conceptually, OSCAR enables a middle ground between univariate activation mapping and unconstrained multivariate decomposition. OSCAR can be used to (1) identify relevant regions whose connectivity reorganizes due to cognitive processing, (2) detect clinically relevant deviations in patient groups, (3) generate hypotheses about compensatory or maladaptive network reconfiguration, and (4) provide region-level readouts that are directly comparable across tasks, datasets, and cohorts. This region-centered framing of OSCAR can add value to analysis of data from causal perturbation approaches like invasive or non-invasive brain stimulation approaches (e.g., transcranial magnetic stimulation,^65^ deep brain stimulation,^66^ or transcranial direct current stimulation,^67^ where localized interventions shift the embedding of both targeted and downstream regions and thereby provide a principled way to map network effects.

Together, these findings demonstrate that OSCAR is able to identify multivariate group differences in BOLD connectivity patterns of brain regions. Compared to perMANOVA, OSCAR showed an advantage in identifying condition-related regions across multiple experimental tasks as well as in a patient cohort. We anticipate that OSCAR will serve as a valuable tool for researchers aiming to map condition-specific changes in FC, whether in task paradigms, in clinical populations, or in perturbation approaches like invasive or non-invasive brain stimulation.

### Limitations of the study

Several limitations should be considered when interpreting our findings. First, several regions previously implicated in specific tasks based on activation analysis (e.g., the IFG for the NWL task) were not detected either by OSCAR or perMANOVA, suggesting task-related activation of these regions may not coincide with large-scale connectivity reorganization. Instead, changes may be confined to a few specific connections and thus remain undetected when focusing on differences in region-to-whole-brain connectivity profiles as done in our analysis. However, we do not see OSCAR as an alternative to traditional activation analyses, but rather as providing complementary insights. Furthermore, OSCAR currently uses whole brain connectivity profiles, which may restrict its sensitivity when the majority of connectivity across states remain unchanged. Restricting OSCAR to refined connectivity profiles could increase its sensitivity. Second, the datasets analyzed in this study were acquired across multiple sites, introducing potential variability related to scanner hardware, acquisition protocols, and other factors.^68^ Such heterogeneity may contribute to systematic differences or subtle site effects. While the use of a standardized preprocessing pipeline (fMRIPrep) helps to reduce such variability, residual site effects cannot be fully ruled out. However, the results here were largely consistent with prior findings reported in the literature. Third, this study employed the Schaefer 200 regions × 17 networks cortical parcellation complemented with subcortical regions from the Melbourne atlas. The choice of parcellation can influence the sensitivity and specificity of detecting FC differences, as the spatial resolution and network definitions determine how finely regional signals are represented. Larger parcels may pool signals across functionally heterogeneous subregions, potentially masking localized effects, whereas smaller parcels increase sensitivity to subtle differences but are more vulnerable to noise. As a result, the specific parcellation scheme employed may bias which regional effects are detectable, and future work should analyze the influence of using different granularities of a given atlas. Relatedly, OSCAR currently identifies regions exhibiting deviating connectivity profiles, but does not determine with which specific networks or sets of regions these connectivity alterations occur. In future work, OSCAR could be extended to assess whether a region’s connectivity changes with predefined networks or specific subsets of regions. Fourth, most tasks analyzed in this study were designed to maximize group-level effects and are, therefore, not ideally suited for detecting individual differences.^69^ Nevertheless, future applications may benefit from relating region-specific OCSVM border distances in individual subjects to behavioral or clinical phenotypes, thereby enabling subject-level inference. Finally, the reliability of FC is strongly dependent on the length of a sequence. The connectomes used to train OSCAR were derived from scans with a length of 6–8 min. Although this is a commonly used scan length, previous research has indicated that reliability can be greatly improved by using sequences of 9–13 min or longer.^70^ Thus, the performance of OSCAR may further benefit from training on data acquired with longer scan durations.

## Resource availability

### Lead contact

Requests for further information and resources should be directed to and will be fulfilled by the lead contact, Jean-Philippe Kroell (j.kroell@fz-juelich.de).

### Materials availability

This study did not generate new unique materials.

### Data and code availability

• Data from AOMIC and HCP EP are publicly available as of the date of publication. The links are listed in the key resources table. Data from the MeMoSlap consortium (OLM and NWL task data) that were used in this study are available upon reasonable request to the lead contact.
• Original code used in this study has been deposited at Sciebo. The link is listed in the key resources table.
• Any additional information required to reanalyze the data reported in this study is available from the lead contact upon request.

## Acknowledgments

This work was supported by the eBRAIN-Health project. eBRAIN-Health has received funding from the 10.13039/100018693European Union’s Horizon Europe research and innovation program under grant agreement no. 101058516 and HORIZON.1.3, grant no. 101147319. This work was also funded by the 10.13039/501100001659German Research Foundation (Research Unit5429/1 [467143400], CRC1315-B03 [327654276], FL379/22-1, FL379/26-1, FL379/34-1, FL379/35-1, FL379/37- 1, ME3161/5-1, and ME3161/6-1).

## Author contributions

J.-P.K., formal analysis, software, validation, visualization, and writing – original draft; M.A., data acquisition and writing – review and editing; H.K., data acquisition and writing – review and editing; V.M., writing – review and editing; L.P., writing – review and editing; M.M., writing – review and editing and data & funding acquisition; A.F., writing – review and editing and data & funding acquisition; S.E., writing – review and editing; K.R.P., conceptualization, software, writing – review and editing, supervision, and funding acquisition.

## Declaration of interests

The authors declare that they have no known competing financial interests or personal relationships that could have appeared to influence the work reported in this paper.

## Declaration of generative AI and AI-assisted technologies in the manuscript preparation process

During the preparation of this work the authors used ChatGPT in order to draft text/coding. After using this tool/service, the authors reviewed and edited the content as needed and take full responsibility for the content of the published article.

## STAR★Methods

### Key resources table

**Table:** 

REAGENT or RESOURCE	SOURCE	IDENTIFIER
**Deposited data**		

Resting state and task fMRI BOLD data	Amsterdam Open MRI Collection (AOMIC)	PIOP1: https://openneuro.org/datasets/ds002785 PIOP2: https://openneuro.org/datasets/ds002790
Resting state fMRI BOLD data	Human Connectome Project for Early Psychosis (HCP-EP)	https://nda.nih.gov/general-query.html?q=query=featured-datasets:Connectomes%20Related%20to%20Human%20Disease
Resting state and task fMRI BOLD data	Research Unit “Modulation of brain networks for learning and memory by transcranial electrical brain stimulation: A systematic, lifespan approach (MeMoSLAP)	the data used in this study is available upon reasonable request to the corresponding author

**Software and algorithms**		

Python code to run OSCAR, perMANOVA, GLM analysis and region-adapted fcMVPA	this paper	https://fz-juelich.sciebo.de/s/7ktx54wTDMgzRef

### Experimental model and study participant details

#### Participants

Functional MRI (fMRI) data from two open datasets were used. The first dataset, AOMIC, consists of three sub datasets - PIOP1, PIOP2, and ID1000 - containing multimodal 3T MRI data from healthy individuals, including structural and fMRI data. Data from PIOP1 (*N*=226, 129 females, mean age 22.2 ± 1.8 years) and PIOP2 (*N*=216, 120 females, mean age 21.9 ± 1.79 years), were used. Here, the RS data from PIOP2 was used as the reference while the Gender-Stroop task (GStroop) from PIOP1 was used as the target condition. The Amsterdam Open MRI Collection (AOMIC) datasets—comprising the population-based “ID1000” and student-focused “PIOP1” and “PIOP2” studies—were collected with approval from the Faculty’s Ethics Committee of the University of Amsterdam (PIOP1 EC number: 2015-EXT-4366, PIOP2 EC number: 2017-EXT-7568). All participants provided written informed consent prior to participation in accordance with the Declaration of Helsinki.

In addition, RS and task fMRI data of the preparatory phase of a larger, multicenter, double-blinded, sham-controlled crossover tDCS study, *Modulation of brain networks for memory and learning* (MeMoSLAP), was used (https://www.memoslap.de/de/forschung/), restricted to imaging sessions where sham stimulation was applied. Data for two different tasks, object-location-memory task (OLM, *N*=20, 10 females; mean age 25 +/- 5.56,^47^) and novel-word-learning task (NWL, *N*=20, 10 females; mean age = 26 +/- 5.86^56^), were taken from two different RU projects, scanned at the same scanner using the same acquisition parameters. In this case, RS data from the AOMIC dataset from PIOP1 and RS data from the AOMIC dataset from PIOP2 were combined for training (*N* = 442), and task differences were assessed separately for each target condition (OLM and NWL). The MeMoSlap study was approved by the medical ethics committee (Registry number BB015/22). The study followed the guidelines of the Declaration of Helsinki, and participants provided written informed consent before inclusion.

We also used data from the Human Connectome Project for Early Psychosis (HCP EP), that includes RS data from patients with early psychosis (*N*=184, 70 female, mean age 23.1 +/- 3.7) and healthy controls that were matched to the psychosis group participants based on sex, age, handedness, and parental socioeconomic status (*N*=68, 24 female, mean age 24.4 +/- 4.3). Here, training relied on RS data from the healthy controls, while patients’ data was used as target condition. The Human Connectome Project Early Psychosis study was approved by the Institutional Review Board (IRB) of Washington University in St. Louis (project 1U01MH109977-01). All participants provided written informed consent prior to participation in accordance with the Declaration of Helsinki.

The ethics proposals for the use and retrospective analyses of the datasets were approved by the Ethics Committee of the Medical Faculty at the Heinrich-Heine-University Düsseldorf (2018-317-RetroDEuA).

In all analyses, subjects in the reference and target conditions were mutually exclusive, and identical data splits were used for OSCAR and perMANOVA.

### Method details

#### Experimental procedure

The GStroop task (PIOP1, AOMIC) probed cognitive conflict and control processes. Participants viewed images of twelve male and twelve female faces paired with either congruent or incongruent gender labels (Dutch words for “man,” “sir,” “woman,” and “lady,” shown in upper or lower case) positioned above each face. Trials followed a randomized event-related design. On each trial, subjects were shown a face-label composite for 0.5 seconds. Stimulus presentation was followed by an inter-stimulus interval ranging between 4 and 6 seconds (in steps of 0.5 seconds). Subjects were always instructed to respond to the gender of the pictured face, ignoring the distractor word, as fast as possible using their left index finger (for male faces) or right index finger (for female faces). The task lasted 490 seconds (245 volumes, TR = 2 s).

The OLM (MeMoSLAP) task involves learning associations between objects (houses on a two-dimensional [2D] map) and their locations through repeated presentations, utilizing both instruction- and feedback-based mechanisms, using a block design with four learning stages (for more details refer to^47^). For the OLM analysis, only data during the six task blocks was used for further analysis.

The NWL task (MeMoSLAP) required learning picture–pseudoword associations across six blocks, each comprising four learning stages. In each learning block, participants were required to identify correct combinations of five object pictures and pseudowords across four learning stages. Pictures were presented twice in each learning stage (10 trials) and were paired with either a correct or incorrect pseudoword (for more details refer to.^56^ For the analysis of the NWL task, only data during the six task blocks was used in the further analysis.

#### Data preprocessing

For AOMIC, preprocessed data using fMRIPrep version 1.4.1^71^ provided via OpenNeuro was used. In brief, data were motion corrected using mcflirt (FSLv5.0.9,^72^) followed by distortion correction by co-registering the functional image to the respective T1 weighted image with inverted intensity^73^ with six degrees of freedom, using bbregister (FreeSurfer v6.0.1). In a following step, motion correction transformations, field distortion correction warp, BOLD-to-T1-weighted transformation and the warp from T1-weighted to MNI were concatenated and applied using antsApplyTransforms^74^ using Lanczos interpolation.^25^

Data from MeMoSLAP was preprocessed with fMRIPrep 24.1.1.^71^ In brief, each BOLD run was corrected for head motion using mcflirt (FSL,^72^). The BOLD reference was co-registered to the corresponding T1-weighted structural image using FreeSurfer’s mri_coreg, refined with FLIRT boundary-based registration (six degrees of freedom^75^). Spatial normalization to MNI152NLin2009cAsym was performed via nonlinear registration using ANTs.^75^ All spatial transforms—including motion correction, susceptibility distortion correction (when available), BOLD-to-T1w alignment, and T1w-to-MNI warps—were combined and applied in a single interpolation step using cubic B-splines.

Although different fMRIPrep versions were used for the AOMIC and MeMoSLAP datasets, both pipelines implement the same core preprocessing framework, including motion correction, anatomical co-registration, spatial normalization, and confound estimation. To minimize potential version-related effects, all downstream analyses were performed using identical post-processing procedures, including confound regression, atlas-based time-series extraction, and functional connectivity calculation.

The HCP EP dataset was preprocessed with the minimal preprocessing HCP pipeline.^76^ The HCP minimal functional preprocessing pipelines consist of correction of gradient-nonlinearity-induced distortion, realignment of the time-series to correct for subject head motion, registration of the fMRI data to the structural data, reduction of the bias field, normalization of the 4D image to a global mean, masking the data with the final brain mask, and the spatial smoothing using a novel geodesic Gaussian surface smoothing algorithm with 2 mm FWHM. Following minimal preprocessing, ICA-FIX^77^ was applied to identify and remove noise components using an automated classifier.

For all datasets, mean white-matter, CSF, and global signals (including their derivatives and squared terms under the full strategy) were regressed out to remove non-neuronal and global physiological noise. Functional time series were band-pass filtered (0.01–0.08 Hz) and extracted from the Schaefer 200×17 cortical atlas and 32 subcortical Melbourne parcellation without any additional spatial smoothing.

#### OSCAR

To create region-specific models, we here propose a new method, One-class SVM-based Connectome Anomaly Recognition (OSCAR), that uses a multivariate density estimation method (OCSVM), and considers region-to-whole brain connectivity patterns (Figure 8). Consider the case where we want to identify differing connectivity patterns between two Groups, A (reference state, e.g. RS) and B (modulated state, e.g. task or patient data). For a region we train a OCSVM^78^ classifier using the group A data to learn the multivariate density describing the connectivity pattern of that region with all other regions. We then apply this OCSVM model to the connectivity pattern of the same region for individuals in both group A and group B. This provides the inliers (individuals whose connectivity pattern conforms with the OCSVM density estimation) and outliers (individuals whose connectivity pattern diverges) for the two groups. A chi^2^ test is then applied to compare the inliner and outlier proportion of the two groups. Group B will have a higher proportion of outliers if it has a different multivariate profile than group A resulting in a low *p*-value. By repeating this procedure for every region and controlling for multiple testing using Bonferroni correction,^79^ we identify regions where the two groups differ.

**Figure 8:**
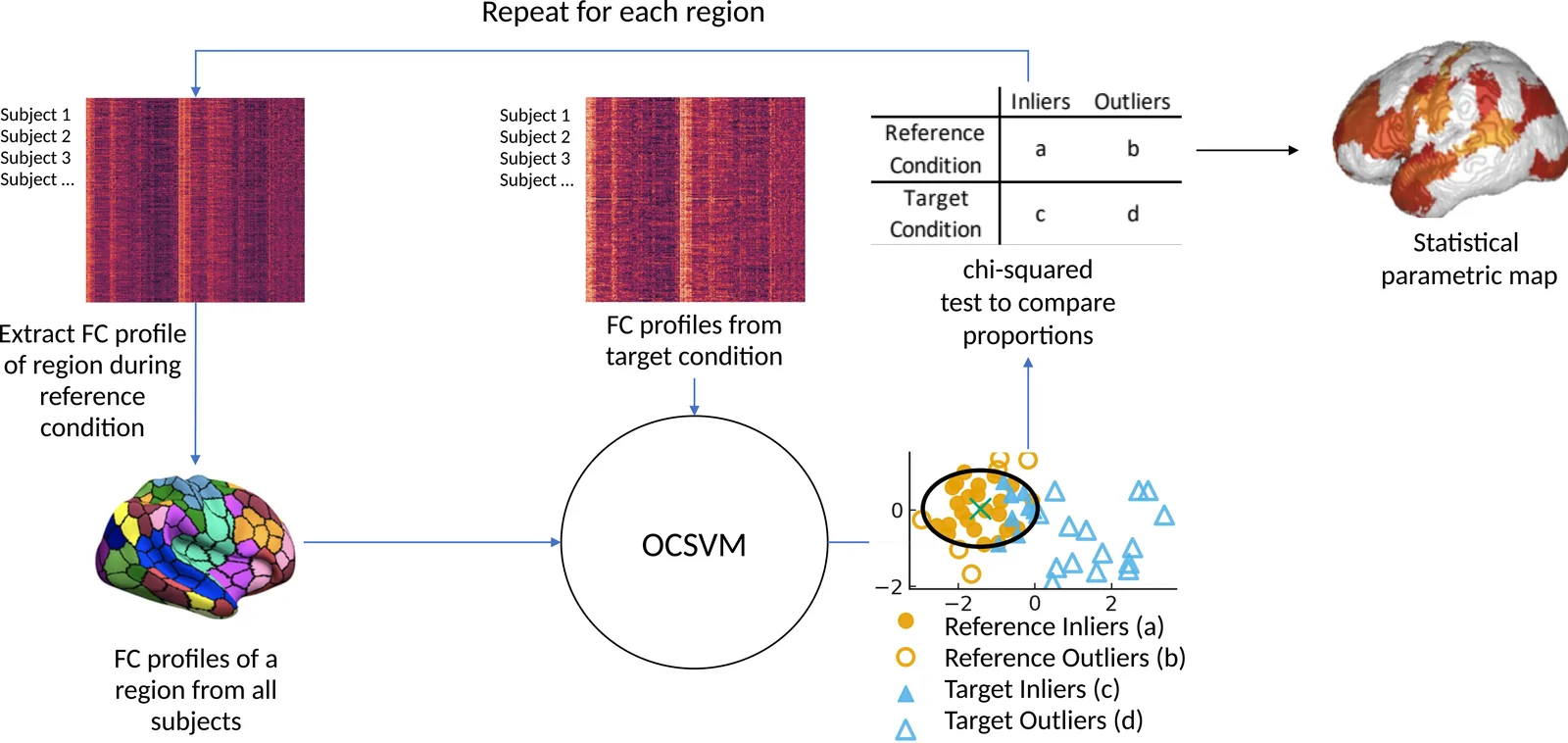
Schematic representation of OSCAR In the first step, the connectivity matrix is calculated for each subject of the RS data, separately. Then, the connectivity profile of a region (i.e., the connectivity of that region with every other region) is taken from all subjects and used to train an OCSVM Classifier. This process is repeated for every region, resulting in one model per region. In the second step, the OCSVM models are applied to connectivity profiles from a target condition (e.g., task- or patient data). Then, the differences between the two groups (RS vs. target condition) are statistically quantified and projected onto a brain map. OSCAR, one-class SVM-based connectome anomaly recognition; OCSVM, one-class support vector machine; FC, functional connectivity.

In this study, we considered the identification of regions affected by a given target condition an outlier detection problem, treating healthy RS as the reference state and aiming to detect outliers within task or patient data. To prepare the input features, we first computed FC matrices using regions defined by the 200-resolution Schaefer parcellation^80^ and the 32 subcortical Melbourne parcellation^81^ in the volumetric space (links to the two GitHub repositories are provided in the supplementary materials). Each resulting 232 × 232 Pearson’s correlation matrix were Fisher r-to-z transformed^82^ and then decomposed into 232 region-specific “connectivity profiles.” The connections representing diagonal values in the FC matrix were removed as they show no variance across subjects. Connectivity profiles from the reference state served as input data for training the OCSVM models. This allowed each OCSVM to model the multivariate density underlying that region’s connectivity with the rest of the brain (in the reference state). We utilized sklearn’s OneClassSVM^78^ with a linear kernel, due to its ability to perform outlier detection in a high dimensional space. The hyperparameter nu (ν) of the OCSVM was optimized using a 5-fold GridSearchCV, searching over the range [0.001, 0.01, 0.025, 0.05, 0.1, 0.25, 0.5]. Νu defines the maximum fraction of training samples allowed to lie outside the decision boundary and the minimum fraction of support vectors that define it. A custom scoring function was used to maximize the number of inliers in the training data. The trained OCSVM models were then applied to connectivity profiles of the target condition individuals. Following the statistical analysis and multiple testing correction, this resulted in a list of regions where the target condition is statistically different from the reference.

#### perMANOVA

We compared OSCAR with perMANOVA based on Anderson.^29^ We selected perMANOVA for comparison as it is designed for detecting multivariate group differences. perMANOVA uses covariance-matrices to perform a distance-based group comparison that tests whether the average multivariate distances between conditions exceed within-condition variability using permutation testing. The following procedure was applied to each region’s connectivity profile. The connections representing diagonal values in the FC matrix were removed as they show no variance across subjects and remaining connections were Fisher r-to-z transformed.^82^ A Euclidean distance matrix was then computed between all pairs of samples, i.e. the FC connectivity profiles of all subjects from two given conditions. Next, a null distribution is generated by permuting the sample assignment to the two conditions multiple times, and the distances were recalculated. A pseudo-F statistic is then calculated by comparing the observed between-group distances to the within-group distances. Finally, the *p*-value is determined by comparing the observed test statistic to the permutation-derived null distribution. We conducted 5000 permutations in this analysis, repeated for each region and controlling for multiple comparisons using Bonferroni correction. In addition, we compared OSCAR to an adapted version of functional connectivity MultiVariate Pattern Analysis (fc-MVPA), a method for detection of multivariate patterns in FC described by Nieto-Castanon.^15^ However, in the datasets used here, fcMVPA did not identify any significant differences (Table S10). This may be due to our region-wise adaptation of the method, which was originally designed for voxel-wise analyses.

#### Validation against prior task-specific activation findings

As there is no single, commonly established method for validating multivariate differences in FC, we relied on two evaluations. First, we quantified the spatial correspondence between the regions detected by a multivariate method (OSCAR or perMANOVA) and regions involved in a respective target condition based on respective task activation analysis or meta analysis. To this end, we used the Euclidean distance of the centroid of the identified regions to the closest (meta-analytic) activation peak as an interpretable similarity metric. This approach provides a reference for assessing whether identified regions lie close to brain areas reliably implicated in the task or, conversely, fall farther away from those regions. For each significant region, only the distance to the closest peak coordinate was considered to quantify its spatial correspondence. Similar spatial correspondence was also used to compare the results of OSCAR and perMANOVA. For the GStroop task, both contrasts of the whole-brain z-value map from the technical validation section of the AOMIC paper were used.^25^ For the OLM task, we used peak coordinates reported by Abdelmotaleb et al.^47^ from significant clusters identified in a whole-brain fMRI analysis conducted on the same subjects included in the present study. The spatial correspondence to both contrasts (learning > control and control > learning) was assessed. For the NWL-task, the peak coordinates from a meta-analysis on lexical learning was used for comparison.^53^ When OSCAR and perMANOVA differed in how many regions they detected, we downsampled the method with more findings to its most significant regions to ensure a fair, equal-sized comparison against task activation.

#### GLM analysis

Region-wise first-level GLMs were fitted to parcellated fMRI time series (TR = 1.0 s, for more details about the parcellation see the method section of this paper). Two task regressors (NWL-task and Control-task) were constructed from condition onsets and durations and convolved with the canonical Glover hemodynamic response function. Low-frequency drifts were modeled using a cosine basis (high-pass filter: 1/128 Hz), and an intercept was included. Six rigid-body motion parameters and the global signal were added as nuisance regressors. First-level models were estimated using an AR(1) noise model.

A first-level contrast comparing conditions (task − control task) was computed for each Region. Subject-level contrast effects were subsequently entered into a second-level one- sample GLM to test whether the mean contrast effect differed from zero across subjects. Results are reported as Region-wise effect estimates, t-statistics, z-scores, and two-sided p- values.

### Quantification and statistical analysis

Statistical analyses were performed as described below. Statistical tests and summary statistics are reported in the corresponding Results and figure legends, where applicable. P-values in the brain plots are presented as −log_10_-transformed values. Exact *p*-values for the region-wise statistical analyses are provided in the corresponding supplemental tables. Distance plots display the minimum Euclidean distance from each region identified by one method to the nearest region identified by the other method or centroid coordinates of the nearest activation peak from the respective studies.

For OSCAR, statistical testing was performed separately for each of the 232 brain regions. For each region, participants from the reference and target conditions were classified as inliers or outliers according to the region-specific OCSVM decision boundary. The numbers of inliers and outliers in the two conditions were compared using a chi-square (χ^2^) test, with n corresponding to the number of participants in each condition (sample sizes are reported in the Participants section). This procedure resulted in one statistical test per region (232 tests in total). P-values were corrected across the 232 regional tests using Bonferroni correction. For perMANOVA, statistical testing was performed separately for each of the 232 brain regions. For each region, the Fisher r-to-z transformed connectivity profiles of participants from the two conditions were used to calculate a Euclidean distance matrix containing the pairwise distances between participants. Here, n represents the number of participants included in the respective conditions, with each participant contributing one region-specific connectivity profile to the analysis. Differences between conditions were assessed using a pseudo-F statistic comparing between-condition and within-condition variability. Statistical significance was determined using 5,000 permutations of the condition labels to generate a null distribution of the pseudo-F statistic. The *p*-value for each region was determined by comparing the observed pseudo-F statistic with its permutation-derived null distribution. This procedure resulted in one statistical test per region (232 tests in total), and *p*-values were corrected across the 232 regional tests using Bonferroni correction. To statistically compare the spatial correspondence of regions identified by OSCAR and perMANOVA with task-related activation findings, we calculated the Euclidean distance between the centroid of each identified region and the nearest task-related activation peak. The distributions of minimum distances obtained for OSCAR and perMANOVA were compared using a two-sided Mann-Whitney U test.

## Supplemental information

Table S1. Brain regions showing significant connectivity-profile differences between resting state and the Gender-Stroop task identified using OSCAR

Table S2. Brain regions showing significant connectivity-profile differences between resting state and the Gender-Stroop task identified using perMANOVA

Table S3. Brain regions showing significant connectivity-profile differences between resting state and the OLM task identified using OSCAR

Table S4. Brain regions showing significant connectivity-profile differences between resting state and the OLM task identified using perMANOVA

Table S5. Brain regions showing significant connectivity-profile differences between resting state and the NWL task identified using OSCAR

Table S6. Brain regions showing significant connectivity-profile differences between resting state and the NWL task identified using perMANOVA

Table S7. Brain regions showing significant connectivity-profile differences between resting state from healthy controls and patients with early psychosis identified using OSCAR

Table S8. Brain regions showing significant connectivity-profile differences between resting state from healthy controls and patients with early psychosis identified using perMANOVA

Table S9. Brain regions and the associated *p* value across all applications using the adapted version of fc-MVPA

Table S10. Region-wise GLM analysis of the NWL-task data
